# Atypical anti-glomerular basement membrane glomerulonephritis in a patient with metastatic melanoma treated with mitogen-activated protein kinase and immune checkpoint inhibitors: a case report

**DOI:** 10.1186/s13256-021-02766-w

**Published:** 2021-04-03

**Authors:** Periklis Kyriazis, Abhinav Tiwary, Jonathan Freeman, Daniel Landry, Gregory Braden

**Affiliations:** 1 Dept. of Internal Medicine, University of Massachusetts Medical School -Baystate, Springfield, MA USA; 2 Division of Nephrology, University of Massachusetts Medical School -Baystate, Springfield, MA USA; 3 Dept. of Pathology, University of Massachusetts Medical School -Baystate, Springfield, MA USA

**Keywords:** Atypical anti-GBM, Immune checkpoint inhibitors, BRAF inhibitors, MEK inhibitors

## Abstract

**Background:**

Immune checkpoint inhibitors and mitogen-activated protein kinase inhibitors have become the standard of care in patients with advanced melanoma bearing V600 mutations. However, little is known about their nephrotoxicity. To date, only two cases of anti-glomerular basement membrane glomerulonephritis after exposure to checkpoint inhibitors have been documented. Herein, we report the first case of a patient with metastatic melanoma who developed linear Immunoglobulin G 3+, Immunoglobulin A 2+, kappa 2+, lambda 1+ anti-glomerular basement membrane glomerulonephritis with negative serology following treatment with checkpoint inhibitors and subsequently mitogen-activated protein kinase inhibitors.

**Case presentation:**

A 58-year-old Caucasian male was referred to our outpatient nephrology clinic with acute kidney injury and proteinuria. He had received three cycles of ipilimumab and nivolumab for recurrent melanoma positive for the BRAF V600E mutation with metastasis to the lungs. Immunotherapy had been discontinued in the setting of severe adverse effects including dermatitis, colitis, and hepatitis. Because of persistent bilateral lung metastases and left pleural metastases, the patient had been initiated on dabrafenib and trametinib until his presentation to our clinic 6 months later. On presentation, his blood pressure was 172/89 mm/Hg and had 2+ edema bilaterally. His creatinine level was 2.4 mg/dL from a previous normal baseline with a urinary protein-to-creatinine ratio of 2 g/g. His urinalysis showed dysmorphic erythrocytes and red blood cell casts. Serologic testing was negative for antineutrophilic cytoplasmic antibodies, proteinase 3 antigen, myeloperoxidase, and anti-glomerular basement membrane antibody. Complement levels were normal. A renal biopsy showed focal crescentic (2 of 15 glomeruli with cellular crescents), proliferative, and sclerosing glomerulonephritis with diffuse linear staining of glomerular capillary loops dominant for IgG (3+), IgA (2+), kappa (2+), and lambda (1+) minimal changes. He was initiated on oral cyclophosphamide and pulse intravenous methylprednisolone followed by oral prednisone for 6 months, which stabilized his renal function until reinitiation of immunotherapy.

**Conclusions:**

Acute kidney injury is an increasingly reported adverse effect of both drug classes, mostly affecting the tubulointerstitial compartment and infrequently the glomerulus. Although the biologic effect of these drugs on immune cells is not entirely understood, it is possible that BRAF-induced podocyte injury in combination with direct T-cell-mediated glomerular injury facilitated by checkpoint inhibitors led to the unmasking of cryptic antigens, loss of self-tolerance, and autoimmunity. More importantly, we show that treatment with corticosteroids and cyclophosphamide was able to improve and stabilize our patient’s renal function until the reinitiation of immunotherapy.

## Introduction

Metastatic melanoma is almost invariably incurable, with a median survival time of less than 1 year and a 3-year survival rate of only 10–15%.[1] The molecular pathogenesis is strongly correlated with constitutive activation of the mitogen-activated protein kinase (MAPK) (RAS-BRAF-MEK-ERK) pathway that excessively signals cells to grow. Melanoma proliferation is attributed to targetable activating BRAF mutations in 50% of cases.[2] In 2014, a combined treatment with dabrafenib, a selective BRAF inhibitor (BRAFi), and trametinib, a selective MEK inhibitor (MEKi) was approved by the Food and Drug Administration (FDA) for the treatment of metastatic melanoma. In 2015, a combination of checkpoint inhibitors (CPI), namely of nivolumab, a monoclonal antibody against programmed cell death receptor-1 (PD-1), and ipilimumab, a monoclonal antibody targeting cytotoxic T-lymphocyte antigen 4 (CTLA-4), was also approved for the same purpose.[3] PD-1 and CTLA-4 are part of a coinhibitory pathway at the CD28 “checkpoint” of T-cell activation. They exist to safeguard T-cell tolerance and protect against autoimmunity. In the setting of malignancy, anti-programmed cell death protein ligand 1 (PD-L1) binds to PD-1, enabling cancer cells to evade host immune surveillance.[4] Anti-CTLA-4 and anti-PD1 checkpoint blockade therapies target distinct tumor-infiltrating T-cell populations to induce tumor rejection.[5]

Despite important clinical benefits, these novel therapies are associated with nephrotoxicity.

BRAF inhibition clinically affects mostly the tubulointerstitial compartment, despite the fact that in murine studies BRAF was shown to be expressed and localized in developing and mature podocytes. Based on published case series, BRAFi can cause acute allergic interstitial nephritis within the first 1–2 weeks or a more subacute tubular injury through direct toxicity within the first 1–2 months of initiation.[6] BRAFi is also implicated in diffuse podocyte injury through BRAF-PCLε1 interactions and nephrin downregulation, leading to nephrotic syndrome.[7]

Similarly, CPI are associated with renal injury, primarily acute interstitial nephritis (AIN) but also glomerulonephropathies, including membranous nephropathy, minimal change disease, focal segmental glomerulosclerosis (FSGS), and granulomatosis with polyangiitis and lupus nephritis. These side effects can take up to 10–12 months to manifest.[8]

Herein, we report the first case of a patient with metastatic melanoma who developed linear Immunoglobulin (Ig) G 3+, IgA 2+, kappa 2+, lambda 1+ anti-glomerular basement membrane glomerulonephritis (anti-GBM GN) with negative serology following treatment with CPI and subsequently MAPKi.

## Case presentation

**Fig. 1:**
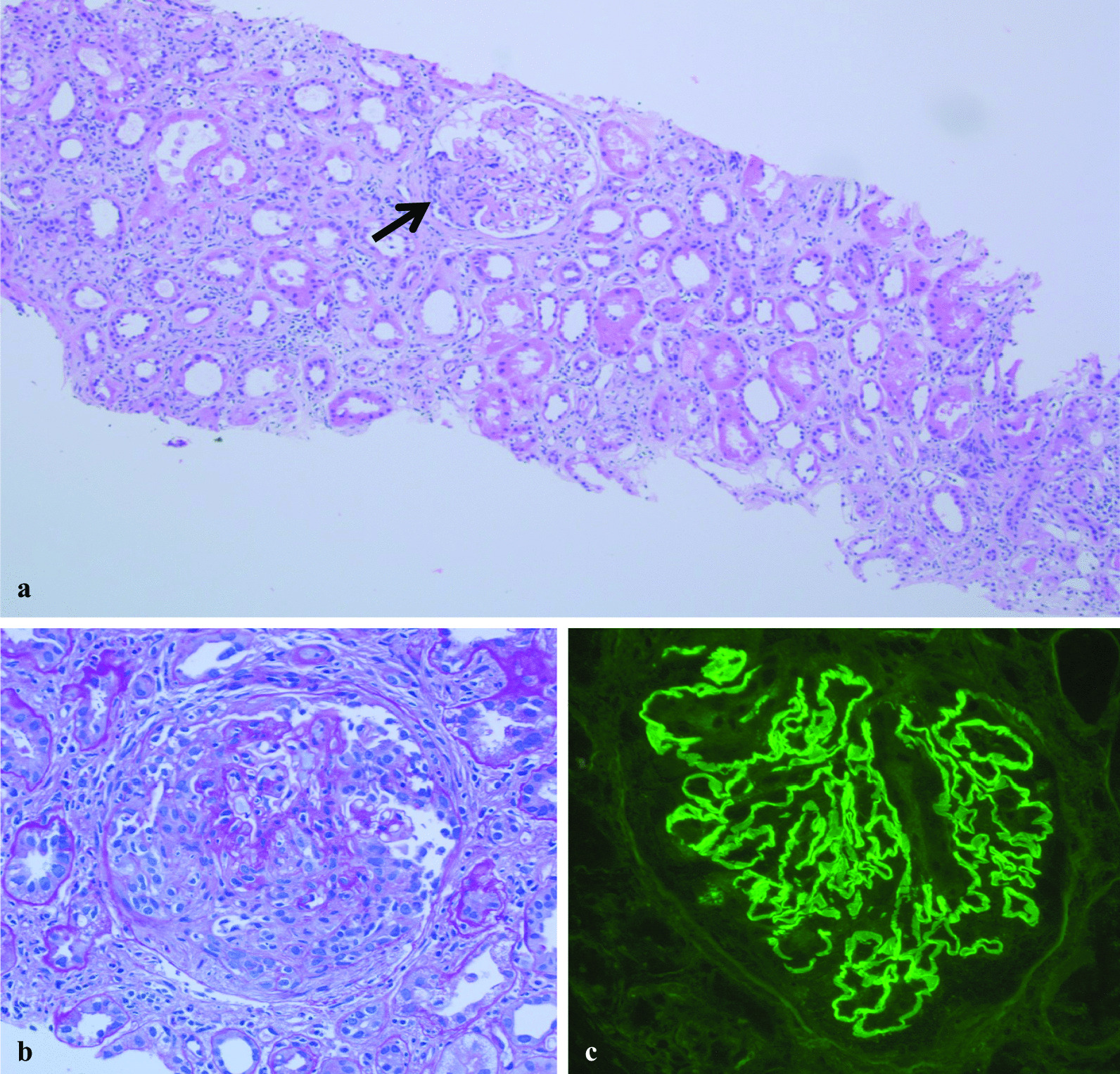
**a** Chronic active interstitial nephritis with focal cellular crescents and linear IgG immunoglobulin deposition. Widespread acute tubular injury with luminal dilatation and flattened epithelium. A glomerulus demonstrates segmental sclerosis and capsular adhesion, possibly and previous crescent (arrow) (hematoxylin and eosin [H&E], original magnification 40×). **b** Glomerulus with cellular crescent (periodic acid–Schiff [PAS], original magnification 200×). **c** Immunofluorescence microscopy demonstrates linear staining for Immunoglobulin G along peripheral capillary loops of all glomeruli (original magnification 200×), also for positive linear IgG3+, IgA 2+, Kappa 2+, Lambda 2 anti-glomerular basement membrane glomerulonephritis (not shown)

A 58-year-old Caucasian male presented with acute kidney injury (AKI) and proteinuria in June 2016. He had originally been diagnosed with melanoma along the right flank in the 1980s that was treated with wide excisional removal. He was followed closely for over three decades with no signs of metastatic disease until he was noted to have a mass near his prior melanoma excisional scar in summer 2014. A biopsy at that time revealed an epithelioid neoplasm with morphology and phenotype highly suggestive of melanoma that was positive for the BRAF V600E mutation. A Positron Emission Tomography/Computed Tomography (PET/CT) at that time showed numerous bilateral pulmonary nodules, and a subsequent right upper lobe wedge resection did confirm metastatic melanoma. In May 2015, he was initiated on ipilimumab (3 mg/kg) and nivolumab (1 mg/kg) every 3 weeks for management of his metastatic melanoma. His course was complicated by grade 3 dermatitis, colitis, and hepatitis, which were treated with courses of prednisone. Therapy was ultimately discontinued in October 2015, after receiving a total of three cycles intermittently, as a result of worsening hepatitis confirmed by liver biopsy. Because of persistent bilateral lung metastases and left pleural metastases, the patient was initiated on dabrafenib 150 mg twice daily and trametinib 2 mg twice daily in December 2015, which he remained on until his presentation to nephrology in June of 2016. He had no personal or family history of chronic kidney disease (CKD). He denied exposure to nephrotoxic agents. He was only taking amlodipine 10 mg for hypertension and had been receiving immunotherapy with dabrafenib and trametinib since December 2015. He denied tobacco use, illicit drug use, and toxic environmental exposure. His blood pressure was 172/89 mm/Hg and had 2+ edema bilaterally. The remainder of his physical examination was normal, and laboratory results indicated a creatinine level of 2.4 mg/dL with a urinary protein-to-creatinine ratio of 2 g/g. His urinalysis showed dysmorphic erythrocytes and red blood cell casts. Renal ultrasonography was normal. Serologic testing was negative for Antineutrophil cytoplasmic autoantibody (ANCA), PR-3, Myeloperoxidase (MPO), and Anti-glomerular basement membrane (anti-GBM) antibody. Complement levels were normal. A renal biopsy was performed in September of 2016 showing focal crescentic (2 of 15 glomeruli with cellular crescents), proliferative, and sclerosing glomerulonephritis with diffuse linear staining of glomerular capillary loops dominant for IgG (3+), IgA (2+), kappa (2+), and lambda (1+) minimal changes (Fig. 1). Ultrastructural examination of three glomeruli demonstrated areas with open capillary loops and preserved foot processes. Other areas demonstrated diffuse effacement of foot processes with variable thickening and wrinkling of glomerular basement membranes. No immune complex disease or tubuloreticular structures were identified.

Repeat anti-GBM testing remained negative, and the patient’s creatinine eventually rose to a peak of 3.8’mg/dL. He had no signs or symptoms of lung hemorrhage. Dabrafenib and trametinib were discontinued, and he was subsequently initiated on oral cyclophosphamide (2 mg/kg/day) and pulse intravenous methylprednisolone (1000 mg daily for 3 consecutive days) followed by 1 mg/kg/day of prednisone. Serum creatinine improved to 2.5 mg/dL, and the active urinary sediment resolved. Immunosuppression with cyclophosphamide was discontinued after 4 months of therapy, and he was weaned off prednisone by 6 months.

The patient was off all immunotherapy for his malignancy, and his renal function remained relatively stable over the ensuing 12 months. By June 2018, a PET/CT of the chest showed evidence of metastatic melanoma to the left upper and medial lobes of the lung. The patient was placed back on nivolumab, which seemed to stabilize his oncologic disease. Unfortunately, 4 weeks after reinitiating nivolumab, his creatinine jumped from 2.8 to 5.8 mg/dL and home hemodialysis was initiated. The patient remains stable on home hemodialysis and was more recently taken off nivolumab and placed back on dabrafenib and trametinib for progressive metastatic pulmonary disease, which has led to significant symptom control.

## Discussion

Acute glomerulonephritis due to anti-GBM antibody is a rare cause of GN, with an incidence of 1:1,000,000, but it also is one of the most aggressive forms of GN. Autoreactive B cells produce antibodies against self-antigens, which in native GBM are sequestered within the quaternary structure of the noncollagenous domains of the triple helix of alpha 3,4,5 chains.[9] Subsequently, glomerular injury occurs through complement activation by the classical pathway, IgG Fc receptor (FcγR) mediated phagocytosis or by direct T-cell-mediated toxicity.[10] Anti GBM-GN typically presents with rapidly progressive glomerulonephritis with or without pulmonary hemorrhage. Its histopathologic hallmark is linear GBM staining for IgG by immunofluorescence (IF) and diffuse crescentic/necrotizing GN affecting more than 50% of glomeruli on light microscopy. *Atypical and rare presentation have also been reported*. Nasr *et al*. [11] in a case series described 20 patients with atypical anti-GBM GN that had a milder, more indolent progressive renal impairment with linear IgG deposition on kidney biopsy, without predominant features of crescentic GN (<40% glomeruli affected) or overt lung hemorrhage. Circulating anti-GBM antibodies were not detected using conventional assays, for which multiple explanations have been proposed, including low-affinity antibodies to the pathogenic substrate, disappearance of circulating antibodies before the resolution of the disease, and cell-mediated rather than humoral immune response.[12]

There are only two cases of typical anti-GBM disease associated with CPI reported so far.[13, 14] The proximate cause for anti-GBM GN in these cases is not yet fully understood, but it is likely that CPI swung the pendulum toward a proinflammatory state conducive to autoimmunity. Specifically, it has been shown that CTL4 blockade depletes Treg populations while enhancing antitumor immunity through the expansion of tumor-infiltrating CD8 T and Th1-like CD4 effector cell populations.[15–17] Persistent antigen stimulation may eventually lead to CD8 cell exhaustion partly due to overexpression of inhibitory receptors such as PD-1, an effect that is abrogated by CPI allowing for reinvigoration of T-cell mechanisms.[5]

Further evidence supporting a link between CPI therapy and anti-GBM disease comes from animal studies. Reynolds *et al*. showed that CD28-B7 checkpoint blockade with an agonistic monoclonal antibody, CTLA4-Ig, that selectively binds to B7.1, reduced autoantibody production and cellular infiltration of glomeruli in an experimental autoimmune rat model of Goodpasture’s disease.[18] Kitching and colleagues further corroborated these findings with an elegant murine study, showing that in humorally mediated anti-GBM GN induced by a foreign antigen, limiting antibody-mediated injury using CTLA4-Fc treatment can attenuate anti-GBM GN.[19]

GN can also be caused by antigen-specific T cells without secreted autoantibodies in experimental anti-GBM nephritis, in which the transfer of activated a3NC1-specific CD4 T cells to naïve rats caused crescentic glomerulonephritis.[20] Autoreactive T cells can subsequently prompt autoreactive B cells, which present the same T-cell epitope of endogenous autoantigens during B-cell epitope spreading, to mount a humoral response. T-cell-mediated glomerular injury may also trigger *de novo* internal immunization of autoantigens released from damaged GBM, which further leads to activation of a group of GBM-specific B cells.[21] Antigen dissemination could have occurred through BRAFi-induced podocyte injury since podocytes are solely responsible for the synthesis and release of the collagen isoform a3a4a5 (type IV) in mature GBM. Perico *et al*. in 2017 reported a case of podocyte injury presenting as nephrotic syndrome after combinatorial treatment with dabrafenib and trametinib. They also demonstrated in *in vitro* experiments that mainly dabrafenib caused profound slit diaphragm disassembly by impairing the BRAF-PLCε1 interactions in podocytes, leading to nephrin downregulation and increased glomerular permeability. Diffuse loss of podocyte cytoarchitecture caused extensive foot process effacement and endothelial injury.[7, 22]

## Conclusions

Taken together, we believe that the use of immune-modulating drugs created a proinflammatory milieu that, in combination with GBM injury and antigen dissemination, provoked loss of peripheral tolerance and autoimmunity. Oncologists and nephrologists should be cognizant of the association of CPI and MAPKi with anti-GBM GN since their use is on the rise. Despite the availability of general guidelines in dealing with anti-GBM GN, further research establishing more specific guidelines is necessary. According to the latest KDIGO guidelines for anti-GBM GN, triple therapy with cyclophosphamide, prednisone, and plasmapheresis is recommended. Kidney transplantation for suitable candidates may be considered after circulating antibodies remain undetectable for a minimum of 6 months. In our case, discontinuation of all immunotherapy agents and treatment with prednisone and cyclophosphamide stabilized our patient’s renal function, until reinitiation of nivolumab caused rapid loss of GFR and dependence on renal dialysis.

## Data Availability

Not applicable

## Abbreviations

ANCA: Antineutrophilic cytoplasmic antibodies
Anti-GBM GN: Anti-glomerular basement membrane glomerulonephritis
CKD: Chronic kidney disease
CPI: Checkpoint inhibitors
CTLA-4: Cytotoxic T-lymphocyte antigen 4
FSGS: Focal segmental glomerulosclerosis
GN: Glomerulonephritis
KDIGO: Kidney disease improving global outcomes
MAPKi: Mitogen-activated protein kinase inhibitors
MPO: Myeloperoxidase
PD-L1: Anti-programmed cell death protein ligand-1
PD-1: Programmed cell death receptor-1
PET/CT: Positron emission spectrometry/computed tomography
PR3: Proteinase 3 antigen
